# Isoflavone-Enriched *Glycine tomentella* Hayata Extract Attenuates Anxiety-like Behavior and Oxidative Stress in Mice via Radical Scavenging Activity

**DOI:** 10.3390/ijms27031560

**Published:** 2026-02-05

**Authors:** Ming-Cheng Tsai, Ming-Chung Lee, Ming-Chong Ng, Yun-Kuan Lin, Pei-Fang Lai, Hsin-Tzu Liu

**Affiliations:** 1 Department of Neurosurgery, Shin-Kong Wu Ho-Su Memorial Hospital, Taipei 111, Taiwan; m000679@yahoo.com.tw; 2 School of Medicine, Fu Jen Catholic University, New Taipei City 242, Taiwan; 3 Department of Life Science, National Taiwan Normal University, Taipei 116, Taiwan; t43006@ntnu.edu.tw; 4 Center for General Education, National Quemoy University, Quemoy 892, Taiwan; 895430096@ntnu.edu.tw; 5 Department of Emergency Medicine, Hualien Tzu Chi Hospital, Buddhist Tzu Chi Medical Foundation, Hualien 970, Taiwan; yklin1129@gmail.com; 6 Department of Medicine, Tzu Chi University, Hualien 970, Taiwan; 7 Department of Medical Research, Hualien Tzu Chi Hospital, Buddhist Tzu Chi Medical Foundation, Hualien 970, Taiwan

**Keywords:** antioxidant capacity, isoflavones, anxiety-like behavior, *Glycine tomentella* Hayata extract

## Abstract

Flavonoids exert antioxidant activity by scavenging free radicals, chelating metals, and modulating antioxidant enzymes. The root extract of *Glycine tomentella* Hayata (GTE), a traditional Chinese medicinal herb contains flavonoids, particularly, isoflavones. However, its neuroprotective effects against anxiety remain unclear. In this study, the effects of GTE on anxiety-like behavior and oxidative stress in male Balb/c mice were investigated. The mice were administered GTE orally once daily for 14 d and subsequently, the anxiolytic-like effects of the extract were observed via elevated plus maze and open field tests. Oxidative stress levels in the treated mice were also measured. The results obtained identified daidzein (9.19 mg/g dry extract) and daidzin (2.95 mg/g dry extract) as the key isoflavones in GTE. Furthermore, free radical scavenging assays confirmed that GTE shows strong antioxidant activity, with an IC50 value of 8.82 μg/mL. It also showed pronounced anxiolytic effects, attenuating reactive oxygen species production in a dose-dependent manner. Mechanistic studies attributed these effects to the estrogenic activity of daidzein, which possibly modulates emotional state via estrogen receptor activation and systemic oxidative stress mitigation. These findings suggest that isoflavone-enriched GTE is a natural resource with potential for use as an antioxidant for mitigating anxiety.

## 1. Introduction

Modern lifestyles, which are generally associated with stress, can contribute to anxiety. While adaptive anxiety is a natural and useful response to environmental stress [1,2] as it increases awareness regarding underlying threats, it can lead to anxiety disorders when prolonged and intrusive [3,4]. At present, the most prevalent mental diseases include generalized anxiety disorder, phobia, and panic disorder. Epidemiological surveys have also suggested that a third of the global population will experience an anxiety disorder during their lifespan [1,5].

Benzodiazepines are frequently used as treatments for anxiety. However, growing concerns regarding tolerance, dependence, and sedation limit their use [6]. Furthermore, existing guidelines recommend selective serotonin reuptake inhibitors and selective serotonin-norepinephrine reuptake inhibitors as first-line medications for anxiety disorders [7]. However, these therapeutic agents are associated with several side effects, including hypertension, bleeding, nausea, and vomiting [8,9,10]. Therefore, prevention strategies targeting progression from normal anxiety to pathological anxiety with minimal side effects are urgently needed.

Anxiety is triggered by a wide range of factors, with stress being the predominant contributor. Multiple studies involving rodent models of chronic and social defeat stress have shown that elevated oxidative stress is associated with anxiety-like behavior [11,12,13]. Oxidative stress has also been implicated in the pathogenesis of anxiety disorders [13,14]. Additionally, several studies have shown that antioxidants, which scavenge free radicals, lowering oxidative stress-induced damage in the brain, can be used to attenuate anxiety [15,16,17]. Similarly, an epidemiological study among Iranian adults revealed a significant negative correlation between dietary total antioxidant capacity and anxiety [18]. Therefore, antioxidants have potential as therapeutic agents against anxiety.

Flavonoids, which are plant-derived natural compounds, have antioxidant properties and show high efficacy in the treatment of emotional disorders. Reportedly, they exert their antioxidant activity by scavenging free radicals, chelating metals, and modulating antioxidant enzyme activity [19,20,21]. Therefore, herbal materials rich in various flavonoids have potential as alternative therapeutic agents for mental illnesses with minimal side effects [22]. The main root of *Glycine tomentella* Hayata, belonging to the Leguminosae family and named “I-Tiao-Gung” in traditional Chinese medicine, is extensively used as a remedy for arthritis and rheumatism in Taiwan. Studies have shown that it is rich in flavonoids, primarily isoflavones [23], which show antioxidative properties via radical scavenging activity and tyrosinase inhibition [24]. Therefore, considering the close association between neuroinflammation and emotional dysregulation, the use of such phenolic antioxidants represents a plausible therapeutic strategy for emotional dysregulation [25,26]. Isoflavones, which are phytoestrogens with estrogenic activity, have also been shown to exert therapeutic effects against psychiatric symptoms in menopausal women [27]. Overall, these previous studies support the hypothesis that plant extracts containing isoflavones may modulate the emotional state of individuals. To verify this hypothesis, the antioxidative and anxiety disorder-mitigating effects of isoflavone-enriched *G. tomentella* Hayata extract (GTE) in mice were investigated. This study may provide valuable insights for developing effective treatments for anxiety disorders with minimal side effects.

## 2. Results

### 2.1. Isoflavone Content of Ultrasound-Obtained GTE

**Figure 1:**
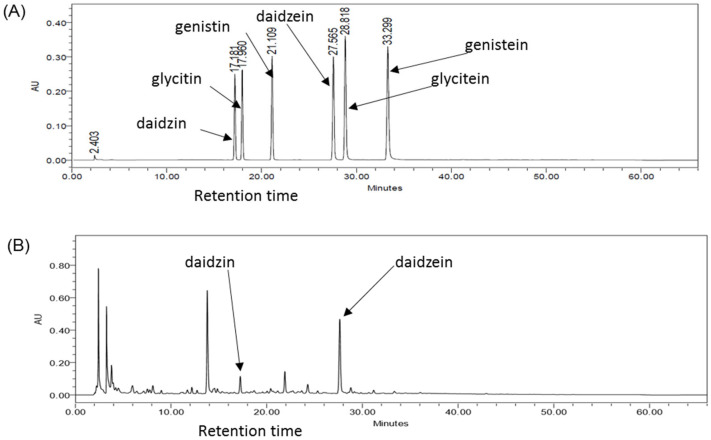
(**A**) High-performance liquid chromatography (HPLC) chromatograms of six isoflavone standards. (**B**) HPLC chromatograms of the aqueous extract of *Glycine tomentella* (GTE).

HPLC (Figure 1) showed the presence of six bio-active isoflavones in GTE, with their relative proportions varying in the order: daidzein (9.19 mg/g dry extract) > daidzin (2.95 mg/g dry extract) > glycitein (0.52 mg/g dry extract) > genistin (0.45 mg/g dry extract) > glycitin (0.43 mg/g dry extract) > genistein (0.15 mg/g dry extract).

### 2.2. GTE Exerts Strong Antioxidant Activity

**Figure 2:**
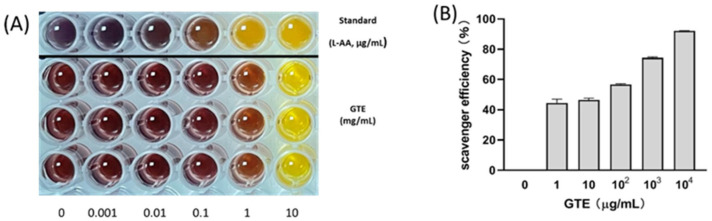
(**A**) 2,2-diphenyl-1-picrylhydrazyl (DPPH) assay results. (**B**) Dose-dependent antioxidant activity of *Glycine tomentella* extract.

GTE exhibited strong antioxidant activity in a dose-dependent manner (Figure 2), showing an IC50 value of 8.82 μg/mL. Furthermore, according to antioxidant activity categories defined by Phongpaichit et al. [28], an IC50 value < 10 μg/mL is indicative of very strong antioxidant activity.

### 2.3. GTE Attenuates Anxiety-like Behavior

**Figure 3:**
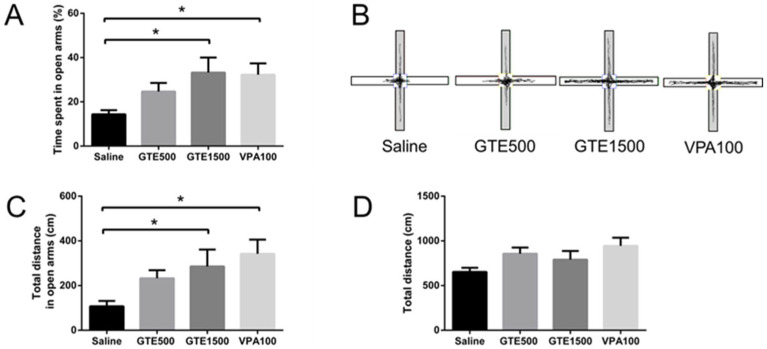
Elevated plus-maze test conducted after the oral administration of an aqueous extract of *Glycine tomentella* (GTE) once daily for 14 d. (**A**) Time spent and (**C**) total distance covered in the open arms of the maze following GTE administration at 1500 mg/kg. (**D**) Total distance covered in the entire maze. (**B**) Trail of mice in the maze at different GTE concentrations. The white and grey areas represent open and closed arms, respectively. Data are presented as mean ± SEM (*n* = 6–10) * *p* < 0.05.

**Figure 4:**
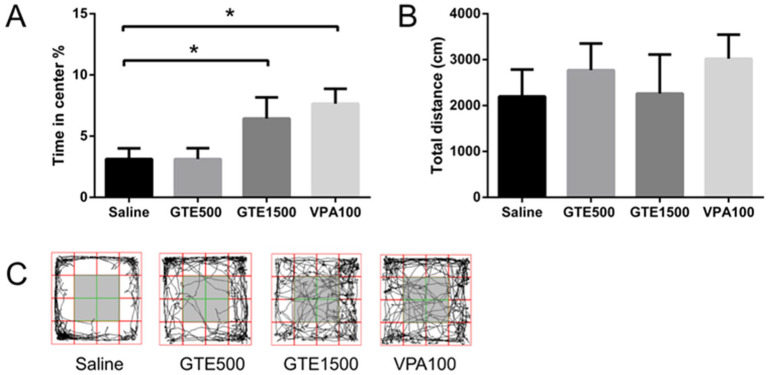
Open field tests conducted after the oral administration of *Glycine tomentella* extract (GTE) once daily for 14 d consecutively. (**A**) Time spent in the central area. (**B**) Total distance traveled in the field. (**C**) Trail of mice in the field. The grey area represents the central area. All data are presented as mean ± SEM (*n* = 6–10); * *p* < 0.05.

Compared with the control (saline) group, the time spent in the open arms of the maze in the EPM test and in the central area of the field in the OF test increased significantly following GTE administration at 1500 mg/kg (Figure 3 and Figure 4). Therefore, GTE mitigated anxiety-like behavior, as the mice showed an increased capacity explore novel environments. However, the results of both behavioral tests showed no significant differences among the treatments in terms of total distance covered, indicating that GTE treatment did not affect the locomotor activity of the test animals.

### 2.4. GTE Exhibited Substantial Antioxidative Effects In Vivo

**Figure 5:**
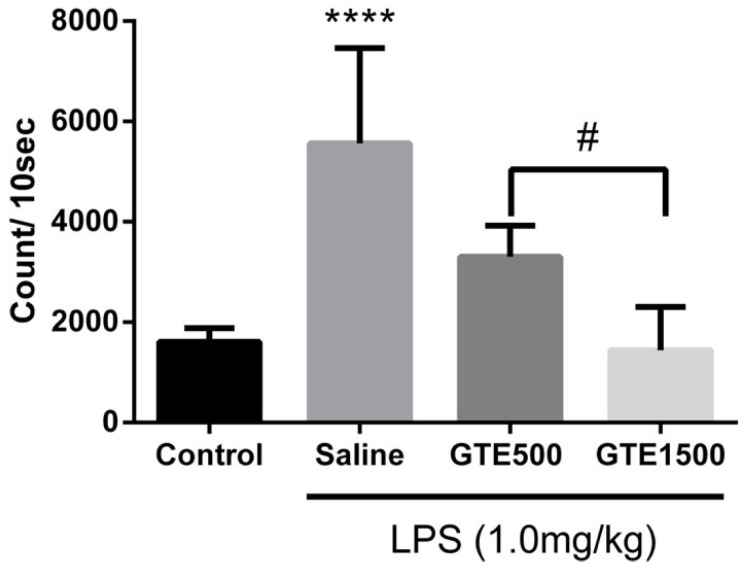
Effect of orally administered *Glycine tomentella* extract treatment (once daily for 18 d consecutively) on lipopolysaccharide-induced reactive oxygen species production in blood. Data are presented as mean ± SEM (*n* = 6–8) **** *p* < 0.001, Saline vs. naïve group; ^#^ *p* < 0.05, GTE500 vs. GTE1500.

A comparison of the naïve group with the saline group showed that LPS induction significantly elevated ROS levels in the blood of mice, leading to oxidative stress, which induced cell damage. However, GTE treatment significantly inhibited LPS-induced ROS production in a dose-dependent manner (Figure 5), demonstrating its substantial effect on antioxidative stress in vivo.

## 3. Discussion

Extraction solvents and methods influence the efficiency of active ingredient extraction from plant materials [29,30]. Kwun et al. reported that conventional extraction using ultrasonication increases the isoflavone content of kudzu extracts [31]. Furthermore, considering that isoflavones are the main active components in “I-Tiao-Gung,” which is predominantly extracted in Taiwan via water extraction, we combined water extraction with ultrasonication for 3 h in this study to improve isoflavone extraction efficiency. The results of HPLC analysis showed daidzein and daidzin contents of 9.19 and 2.95 mg/g dry extract, respectively, significantly higher than those obtained via water extraction only (4.23 and 1.34 mg/g dry extract, respectively) in a previous study [24]. Therefore, combining water extraction with ultrasonication significantly improves the yield of major isoflavones.

Additionally, isoflavones, which are structurally similar to estradiol, are phytoestrogens with estrogenic activity owing to their ability to bind to estrogen receptors (ERs) [32]. ERs are classified as ERα and ERβ, and reportedly, ERβ null mutants show greater levels of anxiety than ERα null mutants, implying that ERβ is implicated in anxiety control [33]. Daidzein, the most abundant isoflavone in GTE, has also been shown to lower anxiety in mice, possibly owing to S-equol (a metabolite of daidzein) binding with nuclear ERβ [34]. Recent studies have also shown that daidzein supplementation significantly ameliorates depressive and anxiety-like behavior in chronic stress models. Mechanistically, this effect is mediated via ERβ activation and upregulation of the extracellular signal-regulated kinase (ERK) pathway and mTOR expression in the hippocampus and cortex, critical pathways for neuroprotection and stress resilience [19,35]. Behavioral evaluation in this study via EPM and OF tests confirmed the anxiolytic effects of GTE. Notably, GTE administration at 1500 mg/kg exerted strong anxiolytic-like activity similar to that of VPA. This observation, at least in part, may be attributed to the high daidzein content of GTE and its subsequent metabolism into S-equol, a selective ligand for ERβ. Regardless, further investigations are required to determine whether the anxiolytic effects of GTE involve S-equol production. It is also necessary to investigate the ERβ-binding activity of S-equol as well as the upregulation of the ERK pathway and mTOR expression in the hippocampus and cortex of the test animals following GTE administration.

Anxiety is associated with oxidative stress, which induces neuroinflammation and, subsequently, anxiety disorders [14]. Therefore, plant-based antioxidants, such as flavonoids, including isoflavones, are potential novel therapeutic agents for anxiety [22,36]. Reportedly, phenolic antioxidants with radical scavenging activity suppress inflammatory responses, potentially via the disruption of redox cycling mechanisms [26]. In this study, radical scavenging assays showed strong antioxidant activity for isoflavone-enriched GTE. Additionally, GTE administration via oral gavage for 18 d consecutively significantly attenuated ROS production in blood, further confirming the antioxidative properties of GTE in vivo. These observations are consistent with the findings of a recent study involving rats with cyclophosphamide-induced hemorrhagic cystitis and bladder hyperactivity, which showed that “I-Tiao-Gung” extract exhibits antioxidative and anti-inflammatory effects [37]. GTE has also been shown to exert anti-inflammatory activity [38], with emerging evidence from recent reviews indicating that dietary flavonoids exhibit potent neuroprotective effects by modulating microglial activation and suppressing pro-inflammatory cytokines in the brain [36]. Therefore, these compounds are increasingly being recognized as therapeutic candidates against neuroinflammation-related mental health disorders [21,39].

Overall, GTE is an accessible and natural source of natural antioxidants with anxiety-mitigating potential. This study is the first to demonstrate that GTE, owing to its high isoflavone content, can alleviate anxiety-like behavior, providing evidence that may facilitate the development of herbal or pharmaceutical treatments for preventing the progression of normal anxiety to anxiety disorders.

## 4. Materials and Methods

### 4.1. Preparation of GTE

*G. tomentella* Hayata was authenticated and deposited as a voucher specimen (Voucher No. TAI-258511) in the National Taiwan University Herbarium, Taiwan. The plant material, cultivated in the Kinmen area of Taiwan, was provided by the Kinmen County Agriculture Research Institute (Kinmen, Taiwan). To prepare the GTE, 1000 g of dried *G. tomentella* Hayata root was ground and extracted with distilled water (4000 mL) at 80 °C under ultrasonication for 3 h. The resulting filtrate was collected, concentrated, and dried via freeze drying to obtain a powder sample, which was then stored at −80 °C. Before use in animal experiments, this final dried extract (GTE) was validated via high-performance liquid chromatography (HPLC).

### 4.2. HPLC Analysis of Isoflavones in GTE

To determine the isoflavone composition of the prepared GTE, HPLC was performed using the Alliance 2695 HPLC system (Waters Corp., Milford, MA, USA), comprising a 2695 separation module (Waters Corp.), 2996 photodiode array detector (Waters Corp.), and ZQ4000 mass spectrometer (Waters Corp.). Furthermore, six isoflavones (daidzin, genistin, glycitin, daidzein, genistein, and glycitein) were targeted and identified by comparing their retention times and co-chromatographic profiles with those of known standards. The concentrations of the identified isoflavones were subsequently determined based on peak area ratios.

### 4.3. Radical Scavenging Assay of GTE Using 2,2-Diphenyl-1-Picrylhydrazyl (DPPH)

The antioxidant activity of GTE was estimated in vitro via DPPH assays. A GTE solution (0.1 mL) was mixed with 0.1 mL of 0.15 mM DPPH (CAS 1898-66-4; Sigma-Aldrich, St. Louis, MO, USA) in methanol. After shaking vigorously to ensure proper mixing and allowing to stand in the dark for 30 min, absorbance measurements were performed at 517 nm, and radical scavenging efficiency was calculated according to Equation (1).

(1) $$\text{Radical}\;\text{scavenging}\;\text{efficiency}\left(\%\right)=\frac{{1-\text{Absorbance}}_{\text{sample}}}{{\text{Absorbance}}_{\text{control}}}$$

The assays were performed in triplicate using L-ascorbic acid as the standard. A graph of scavenging activity against GTE concentration was also generated, and the IC50 value of GTE, defined as the GTE concentration required to induce a 50% decline in the initial DPPH absorbance, was calculated via nonlinear regression using GraphPad Prism 8.0 (GraphPad Software Inc., La Jolla, CA, USA).

### 4.4. Housing Conditions for the Test Animals

Behavioral experiments were conducted using male Balb/c mice (age, 8 weeks; weight, 20–30 g) purchased from BioLASCO (Taiwan Co. Ltd., Taipei, Taiwan). All the animals were housed in cages (*n* = 5 per cage) in an air-conditioned room at 25 °C under a 12 h/12 h light-dark cycle, with light from 06:00 to 18:00. Food and water were provided ad libitum, and the mice were left undisturbed for at least 1 week before the behavioral tests. Additionally, the animal tests were approved by the Institutional Animal Care and Use Committee of National Taiwan Normal University, Taipei, Taiwan (approval number 105043).

### 4.5. Evaluation of Anxiety-like Behavior

Anxiety-like behavior was evaluated via elevated plus-maze (EPM) and open field (OF) tests, which are standard behavioral assays for assessing anxiety in rodents [40,41]. The mice were randomly divided into four groups: the saline, GTE500, GTE1500, and VPA100 groups, administered saline (10 mL/kg), 500 mg/kg GTE, 1500 mg/kg GTE, and 100 mg/kg valproate (CAS 1069-66-5; Sigma-Aldrich), respectively. The mice in the saline and GTE groups were orally administered the respective treatments once daily for 14 d consecutively, after which behavioral tests were performed on day 15. Furthermore, mice in the VPA100 group were intraperitoneally (i.p.) administered valproate 30 min before the behavioral test. The VPA100 group served as a positive control. Furthermore, the same mouse cohorts were used for EPM and OF tests, with an interval of at least 4 h between the two tests to minimize potential interference.

### 4.6. EPM Tests

The maze consisted of two open arms (40 × 10 cm) without walls and two closed arms (40 × 10 cm) with 50 cm high walls. Each maze, with the open arms perpendicular to the closed arms, was positioned 50 cm above the floor, and each mouse was placed at its center. Thereafter, the behavior of the mouse within 5 min was videotaped. Subsequently, a commercially available video tracking software (SMART 3.0, Panlab, Barcelona, Spain) was used to analyze the recordings, focusing on the time spent in the open arms and total distance covered in the open and closed arms of the maze.

### 4.7. OF Tests

OF tests were conducted in a cubic acrylic chamber (42 × 42 × 36 cm) under low illumination (30 lx). The floor of the chamber was divided equally into 16 squares, with the central part of the floor of the chamber (comprising four squares in the middle) defined as the central area, whereas the remaining squares were defined as peripheral areas. The behavior of each mouse in the chamber was videotaped for 10 min. Thereafter, the time spent in the center of the chamber and the total distance covered by each mouse were determined using SMART 3.0. (Panlab).

### 4.8. Antioxidation Capacity Assessment in Whole-Blood

To assess the antioxidation capacity of GTE, mice were divided randomly into four groups, namely, the naïve, saline (administered saline at 10 mL/kg), GTE500 (administered GTE at 500 mg/kg), and GTE1500 (administered GTE at 1500 mg/kg) groups. The saline, GTE500, and GTE1500 groups were the same mouse cohorts used in the behavioral tests. Saline and GTE were administered orally once daily for 18 d consecutively, and 1 h after administration on the last day, all the mice in the three treatment groups were injected lipopolysaccharide (LPS, serotype 055: B5, 10 mg/kg, i.p.; Sigma-Aldrich) to induce reactive oxygen species (ROS) production. Finally, 24 h after LPS injection, blood samples were collected and analyzed. Blood samples from naïve mice were also analyzed.

Antioxidant activity in the blood samples was measured using a chemiluminescence (CL) analysis system. The CL signal emitted from a 200 µL blood sample detected using the ultrasensitive CL analyzer (CLD-110; Tohoku Electronic Industrial, Sendai, Japan) for 1 min served as the baseline level. Subsequently, 500 µL of lucigenin, which reacts with various ROS to trigger luminescence, was added to the blood samples and continuous signal recording was performed. The total CL count for each sample was then estimated by integrating the area under the curve of the signal over 300 s, and the results obtained were presented as CL counts per 10 s.

### 4.9. Statistical Analysis

All statistical analyses were performed using GraphPad Prism v8.0 (GraphPad Software Inc.), and data were presented as mean ± standard error of the mean (SEM). Group differences were determined via analysis of variance followed by post hoc Tukey honestly significant difference tests, and statistical significance was set at *p* < 0.05.

## Data Availability

The data presented in this study are available on request from the corresponding author.

## Abbreviations

The following abbreviations are used in this manuscript:

CL	Chemiluminescence
EPM	Elevated Plus Maze
ERs	Estrogen Receptors
ERK	Extracellular Signal-Regulated Kinase
GTE	*Glycine tomentella* Aqueous Extract (or Water extract of *Glycine tomentella* Hayata)
HPLC	High Performance Liquid Chromatography
i.p.	Intraperitoneally
LPS	Lipopolysaccharide
OF	Open Field
ROS	Reactive Oxygen Species
SEM	Standard Error of the Mean
